# Supplementary material for: A Multiplex Serological Assay to Evaluate the Antibody Responses to a Set of Plasmodium falciparum Antigens and Their Protective Role Against Malaria in Children Aged 1.5 to 12 Years Living in a Highly Seasonal Malaria Transmission Area of Burkina Faso
Source: Vaccines (Basel). 2025 Oct 24;13(11):1091. doi: 10.3390/vaccines13111091 (PMC12656952; doi:10.3390/vaccines13111091)
Supplement: Supplementary file 1 [file vaccines-13-01091-s001.zip › vaccines-3734952-supplementary.pdf]

Supplementary Material

Supplementary Table S1. Laboratory information and references for antigen panel [18].

**Antigen (Alias)**	**Full name and information**	**Primary Expression Stage in Human Host**	**Notes**
CSP	Circumsporozoite protein NANP repeat	Pre-erythrocytic (sporozoite)	Major sporozoite surface protein; key vaccine target (liver stage)
MSP2 CH150/9	Merozoite surface protein 2	Erythrocytic (merozoite)	Surface protein variant on the merozoite, highly variable
MSP2 Dd2	Merozoite surface protein 2	Erythrocytic (merozoite)	Another allelic form of merozoite surface protein 2
AMA1	Apical membrane antigen 1, N terminal region	Erythrocytic (merozoite)	Involved in both liver and blood stage cell invasion
Etramp5 Ag1	Early transcribed membrane protein 5, antigen 1	Erythrocytic (early ring stage)	Marker of recent exposure; expresses during early blood stage
HSP40 Ag1	Heat shock protein 40	Erythrocytic (trophozoite/ring)	Heat shock protein, essential for survival in blood stages
EBA175	Erythrocyte binding antigen 175, region III-V	Erythrocytic (merozoite)	Enables red blood cell invasion (glycophorin A pathway)
Etramp4 Ag2	Early transcribed membrane protein 4, antigen 2	Erythrocytic (early ring stage)	Expressed during early blood infection
GEXP18	Gametocyte-exported protein 18	Erythrocytic (asexual stages)	Marker of recent exposure and possibly early gametocytes
SEA1	Schizont egress antigen 1	Erythrocytic	Protects against parasite egress, potential blood-stage vaccine target
EBA181	Erythrocyte binding antigen 181, region III-V	Erythrocytic (merozoite)	Red blood cell binding protein
RH5.1	Reticulocyte binding protein homologue 5	Erythrocytic (merozoite)	Essential for red blood cell invasion in all isolates
SBP1	Skeleton binding protein 1	Erythrocytic (trophozoite/schizont)	Exported to membrane of infected red blood cells
Hyp2	Exported putative protein	Erythrocytic (early blood stages)	Exported protein with less-defined function
GLURP R2	Glutamate-rich protein R2 region	Erythrocytic (trophozoite to gametocyte)	Immunogenic, exposure marker, broad expression in blood stage
Rh4.2	Reticulocyte binding homologue 4	Erythrocytic (merozoite)	Involved in alternative invasion pathways
